# Gangrene of the Lung

**Published:** 1844-10-05

**Authors:** 


### GANGRENE OF THE LUNG.

Dr. Tinniswood narrates in the London and Edinburg Medical Journal the case of a woman 31 years of age, who had long previously presented symptoms of tubercles in the lungs, and who ultimately recovered under his care after the destruction of a considerable part of that organ by gangrene, from inflammation set up in the neighborhood of a large tubercular deposit, and obliterating the vessels of a portion of the lung—the walls of the cavity having afterwards collapsed, and the greater portion of the lung becoming impervious.

